# The effects of tumor necrosis factor-α (TNF-α) rs1800629 and rs361525 polymorphisms on sepsis risk

**DOI:** 10.18632/oncotarget.22824

**Published:** 2017-11-30

**Authors:** Yixin Zhang, Xiaoteng Cui, Li Ning, Dianjun Wei

**Affiliations:** ^1^ Department of Clinical Laboratory, The Second Hospital of Tianjin Medical University, Tianjin 300211, PR China; ^2^ School of Medical Laboratory, Tianjin Medical University, Tianjin 300070, PR China; ^3^ Department of Biochemistry and Molecular Biology, School of Basic Medical Sciences, Tianjin Medical University, Tianjin 300070, PR China

**Keywords:** tumor necrosis factor-α, single nucleotide polymorphisms, sepsis, rs1800629, rs361525

## Abstract

This meta-analysis of 23 eligible articles comprehensively and quantitatively evaluated the effects of tumor necrosis factor-α (*TNF-α*) rs1800629 and rs361525 polymorphisms on sepsis risk. We found that *TNF-α* rs1800629 was associated with increased sepsis risk in the overall population in four genetic models, including A vs. G (*P*<0.001, odds ratio (OR)=1.32), GA vs. GG (*P*<0.001, OR=1.46), GA+AA vs. GG (*P*<0.001, OR=1.46), and carrier A vs. carrier G (*P*<0.001, OR=1.32). Subgroup analyses showed a similar result for Asian patients (all *P*<0.05, OR>1). *TNF-α* rs361525 was also associated with increased sepsis risk in Asian patients in the four genetic models (all *P*<0.05, OR>1). Begg’s and Egger’s tests excluded large publication bias, and sensitivity analysis indicated stable results. Our results suggest that the G/A genotype of *TNF-α* rs1800629 and rs361525 increases sepsis risk in an Asian population.

## INTRODUCTION

Sepsis consumes considerable health care resources and has a high mortality rate, especially in elderly patients and in infants born pre-term or with low birth weights [1, 2]. Lack of early detection is implicated in the high incidences of severe sepsis and septic shock [3, 4]. Thus, sepsis-related biomarkers and risk factors must be identified to improve early detection rates. Recent studies have addressed associations between various gene SNPs (single nucleotide polymorphisms) and sepsis risk. Sepsis risk was associated with *TLR4* (toll like receptor 4) SNPs, rs4986790 and rs4986791 [5], but not the *SERPINE1* [Serpin Peptidase Inhibitor, Clade E (Nexin, Plasminogen Activator Inhibitor Type 1), Member 1] rs1799768 polymorphism [6].

TNF-α (tumor necrosis factor-α) is important for normal body functions, but is also implicated in some disease mechanisms, including sepsis, diabetes mellitus, and cancer [7-11]. There are several known SNPs within the *TNF-α* gene, including rs1800629, rs361525, rs1800630, and others [12]. Associations between *TNF-α* SNPs and sepsis risk are still uncertain. *TNF-α* rs1800629 was reported as a sepsis risk factor in severely injured North Indian patients [13], critically ill Japanese patients [14], the Chinese Han population [15], and Turkish children [16]. However, *TNF-α* rs1800629 was also negatively correlated with sepsis susceptibility in preterm infants in Germany [17] and low-birth-weight infants in Hungary [18]. We did not obtain data from genome wide association studies (GWAS) of sepsis-associated SNPs. Thus, our meta-analysis is a relatively objective evaluation of *TNF-α* SNPs in sepsis risk. Our analysis focused on the genetic relationship between sepsis risk and the rs1800629 and rs361525 polymorphisms within the *TNF-α* promoter region, in that sufficient data was only obtained for the meta-analysis of rs1800629, rs361525 polymorphisms, after our data extraction.

## RESULTS

### Eligible studies

**Figure 1:**
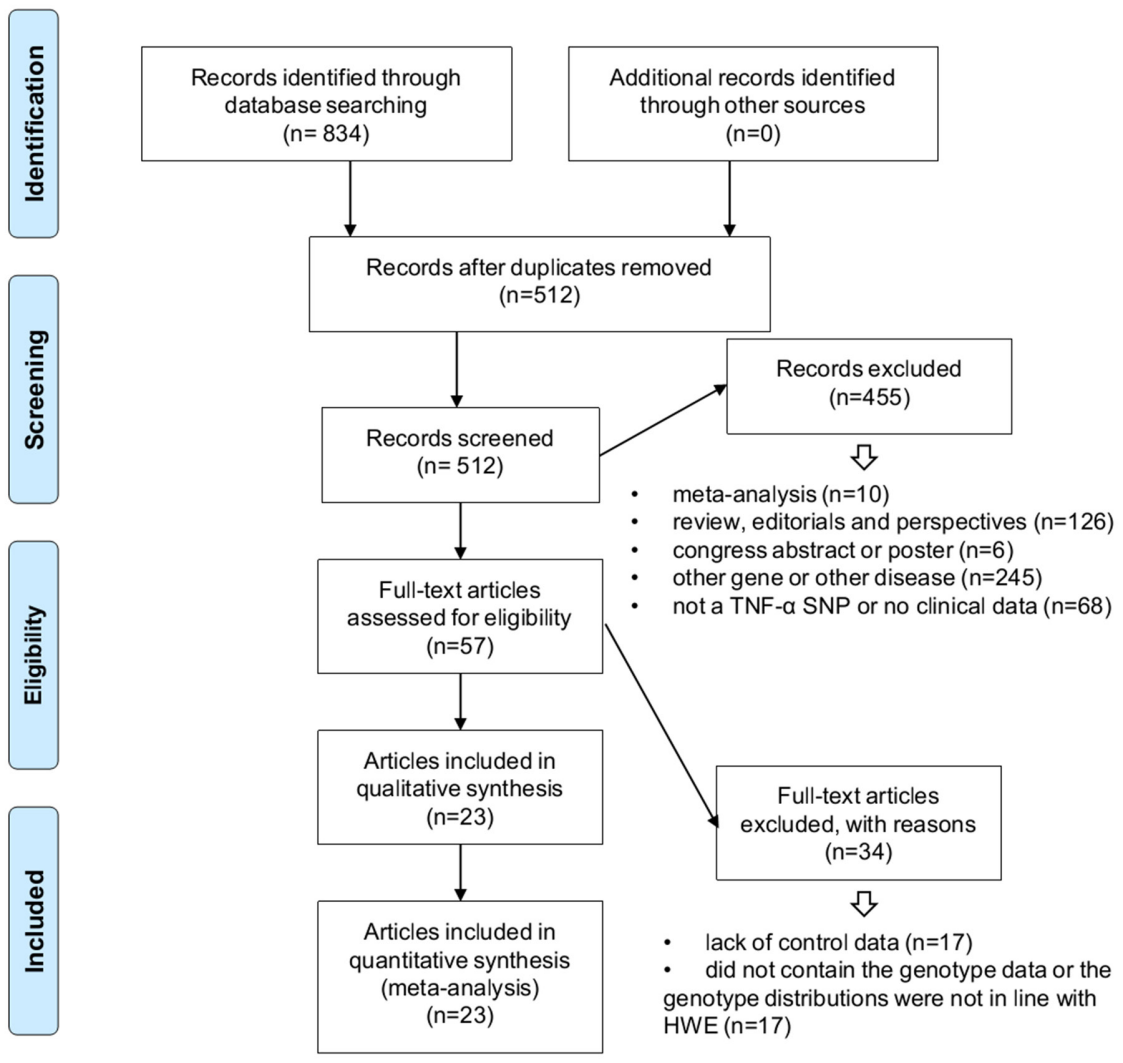
Records identification and study inclusion

We identified a total of 834 records by searching six online databases, including PubMed, WOS (Web of Science), EMBASE (Excerpta Medica Database), CNKI (China National Knowledge Infrastructure), WANFANG, and Scopus, during April 2017 (Figure 1, Supplementary Table 1). We included 23 articles that fit our inclusion/exclusion criteria in our meta-analysis [13-35]. Case/control group characteristics and genotype frequencies are shown in Table 1 and Supplementary Table 2. The 23 articles included 15 high quality studies (NOS score >6 [14-19, 22, 24, 25, 27, 29, 30, 33-35]) and eight medium quality studies (NOS=5 [21, 23, 28]; NOS=6 [13, 20, 26, 31, 32]).

**Table 1:** Characteristics of case-control studies included in this meta-analysis

First author, year	Ethnicity	SNP	Case	Control	NOS	Genotyping assay
Azevedo, 2012 [19]	Caucasian	rs1800629	439	564	7	TaqMan “Assay by Design” system
Balding, 2003 [20]	Caucasian	rs1800629	183	389	6	PCR-RFLP
Davis, 2010 [21]	Caucasian	rs1800629	28	53	5	Taqman SNP allele discrimination assay
Dou, 2007 [22]	Asian	rs1800629	45	60	9	PCR-RFLP
Duan, 2011 [23]	Asian	rs1800629	131	174	5	PCR-RFLP
Fu, 2016 [24]	Asian	rs1800629	115	108	7	PCR-RFLP
		rs361525	115	108	7	PCR-RFLP
Gordon, 2004 [25]	Caucasian	rs1800629	212	354	8	PCR-RFLP
		rs361525	205	354	8	End-labeled allele-specific probe hybridisation.
Gupta, 2015 [13]	Asian	rs1800629	25	89	6	PCR-SSP
		rs361525	25	89	6	PCR-SSP
Majetschak, 2002 [26]	Caucasian	rs1800629	14	56	6	Real-time PCR assay with specific fluorescence-labeled hybridization probes
Mira, 1999 [27]	Caucasian	rs1800629	81	78	7	DGGE analysis
		rs361525	59	72	7	DGGE analysis
Nakada, 2005 [14]	Asian	rs1800629	86	214	7	PCR-RFLP
O'Keefe, 2002 [28]	mixed	rs1800629	37	115	5	Pyrosequencing/PCR-RFLP
		rs361525	37	114	5	Pyrosequencing
Peres, 2012 [29]	Caucasian	rs1800629	166	214	8	PCR-RFLP
Phumeetham, 2012 [30]	Asian	rs1800629	66	101	8	PCR-RFLP
Schaaf, 2003 [31]	Caucasian	rs1800629	28	50	6	PCR-RFLP
Schueller, 2006 [17]	Caucasian	rs1800629	67	233	7	PCR-RFLP
Sipahi, 2006 [16]	Caucasian	rs1800629	53	77	7	PCR-RFLP
Sole, 2010 [32]	Caucasian	rs1800629	320	1152	6	Rapid cycle real-time PCR
		rs361525	320	1172	6	Rapid cycle real-time PCR
Song, 2012 [15]	Asian	rs1800629	802	600	9	gene sequencing
		rs361525	803	598	9	gene sequencing
Tian, 2015 [33]	Asian	rs1800629	32	50	9	PCR-RFLP
		rs361525	32	50	9	PCR-RFLP
Treszl, 2003 [18]	Caucasian	rs1800629	33	35	7	PCR-RFLP
Yu,B, 2003 [34]	Asian	rs1800629	40	100	9	PCR-RFLP
Yu,D, 2007 [35]	Asian	rs1800629	56	60	9	PCR-RFLP

SNP: single nucleotide polymorphisms; NOS: Newcastle-Ottawa Scale; PCR-RFLP: polymerase chain reaction-restriction fragment length polymorphism; PCR-SSP: polymerase chain reaction-sequence specific primer; DGGE: denaturing gradient gel electrophoresis.

### *TNF-α* rs1800629 meta-analysis

We enrolled 27 case-control studies with 3,404 cases and 5,973 controls [13-35] in our *TNF-α* rs1800629 meta-analysis (Table 2). Sepsis risk was increased in the case group in four genetic models: A vs. G (*P* value from association test <0.001, odds ratio (OR)=1.32, 95% confidence interval (CI) =1.05–1.65); GA vs. GG (*P*<0.001, OR=1.46, 95% CI=1.19–1.79); GA+AA vs. GG (*P*<0.001, OR=1.46, 95% CI=1.20–1.78); carrier A vs. carrier G (*P*<0.001, OR=1.32, 95% CI=1.14–1.54), but not other models (all *P*>0.05), compared with controls. This suggested that the *TNF-α* rs1800629 G/A genotype was associated with sepsis risk in the overall population.

**Table 2:** Genetic relationship between *TNF-α* rs1800629 and sepsis risk

Comparison	Subgroup	Sample size		Association test		
		Studies	Case/control	z	*P-*value	OR (95% CI)
**A vs. G**	overall	27	3,404/5,973	3.88	**<0.001**	1.32 (1.05–1.65)
	PB	17	2,388/3,003	2.68	**0.007**	1.41 (1.10–1.80)
	HB	9	796/1,818	2.56	**0.010**	1.44 (1.09–1.91)
	Caucasian	15	1,883/4,191	1.87	0.062	-
	Asian	11	1,484/1,667	3.86	**<0.001**	1.88 (1.36–2.59)
	Sepsis	3	431/668	0.30	0.766	-
	Severe sepsis	9	903/2,934	2.20	**0.027**	1.55 (1.05–2.29)
	Septic shock	6	450/2,203	0.60	0.549	-
**AA vs. GG**	overall	20	3,047/5,320	1.89	0.058	-
	PB	12	2131/2,517	1.51	0.132	-
	HB	7	696/1,651	1.21	0.228	-
	Caucasian	12	1,783/4,023	0.79	0.430	-
	Asian	7	1,227/1,182	2.20	**0.028**	2.25 (1.09–4.63)
	Sepsis	2	403/650	1.26	0.208	-
	Severe sepsis	7	836/2,801	1.13	0.257	-
	Septic shock	6	450/2,203	0.44	0.657	-
**GA vs. GG**	overall	27	3,404/5,973	3.67	**<0.001**	1.46 (1.19–1.79)
	PB	17	2,388/3,003	2.34	**0.019**	1.42 (1.06–1.89)
	HB	9	796/1,818	2.84	**0.005**	1.57 (1.15–2.15)
	Caucasian	15	1,883/4,191	1.64	0.101	-
	Asian	11	1,484/1,667	3.74	**<0.001**	1.96 (1.38–2.78)
	Sepsis	3	431/668	1.04	0.298	-
	Severe sepsis	9	903/2,934	1.89	0.059	-
	Septic shock	6	450/2,203	0.57	0.566	-
**GA+AA vs. GG**	overall	27	3,404/5,973	3.79	**<0.001**	1.46 (1.20–1.78)
	PB	17	2,388/3,003	2.52	**0.012**	1.44 (1.08–1.91)
	HB	9	796/1,818	2.70	**0.007**	1.55 (1.13–2.12)
	Caucasian	15	1,883/4,191	1.74	0.082	-
	Asian	11	1,484/1,667	3.72	**<0.001**	1.95 (1.37–2.78)
	Sepsis	3	431/668	0.76	0.448	-
	Severe sepsis	9	903/2,934	2.06	**0.039**	1.55 (1.02–2.34)
	Septic shock	6	450/2,203	0.57	0.572	-
**AA vs. GG+GA**	overall	20	3,047/5,320	1.52	0.128	-
	PB	12	2131/2,517	1.43	0.152	-
	HB	7	696/1,651	0.81	0.420	-
	Caucasian	12	1,783/4,023	0.53	0.594	-
	Asian	7	1,227/1,182	1.95	0.051	-
	Sepsis	2	403/650	1.35	0.176	-
	Severe sepsis	7	836/2,801	1.09	0.278	-
	Septic shock	6	450/2,203	0.49	0.621	-
**carrier A vs. carrier G**	overall	27	3,404/5,973	3.60	**<0.001**	1.32 (1.14–1.54)
	PB	17	2,388/3,003	2.41	**0.016**	1.33 (1.05–1.67)
	HB	9	796/1,818	2.60	**0.009**	1.35 (1.08–1.70)
	Caucasian	15	1,883/4,191	1.96	0.050	-
	Asian	11	1,484/1,667	3.90	**<0.001**	1.75 (1.32–2.31)
	Sepsis	3	431/668	0.33	0.742	-
	Severe sepsis	9	903/2,934	2.60	**0.009**	1.30 (1.07–1.58)
	Septic shock	6	450/2,203	0.11	0.909	-

PB: population-based control; HB: hospital-based control; OR: odd ratio; CI: confidence interval; -: ORs (95% CIs) not provided for *P*_association_ >0.05.

**Figure 2:**
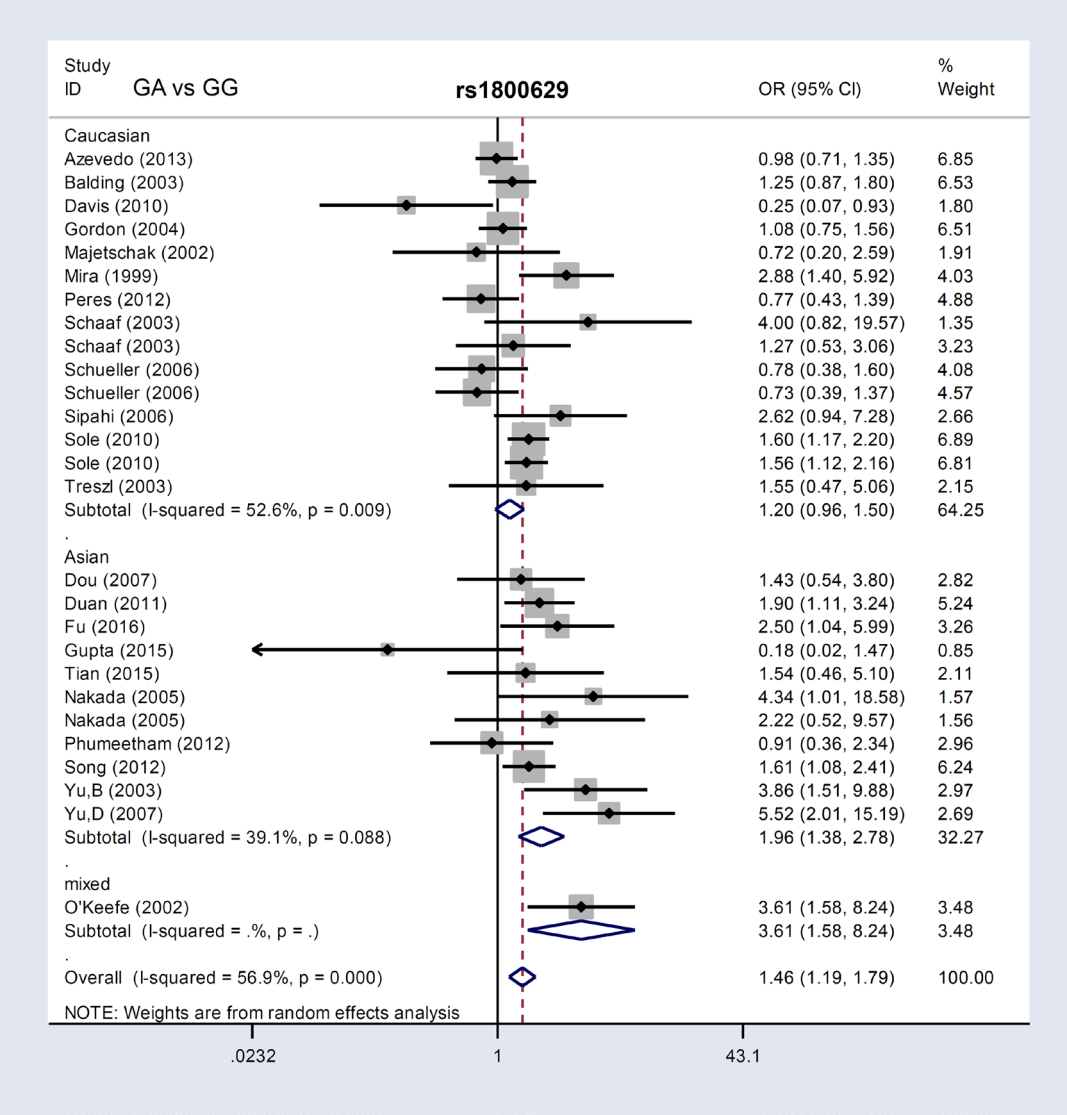
*TNF-α* rs1800629 subgroup analysis based on ethnicity using the GA vs. GG genetic model

Next, we performed meta-analyses stratified as follows: PB (population-based)/HB (hospital-based), Caucasian/Asian, and sepsis/severe sepsis/septic shock. The PB, HB, and Asian patient groups differed from controls in all four models (A vs. G, GA vs. GG, GA+AA vs. GG, carrier A vs. carrier G) (Table 2, *P*<0.05, OR>1). These results showed a positive correlation between the *TNF-α* rs1800629 G/A genotype and sepsis risk in the Asian population. Additionally, in an analysis of eight articles [13, 15, 26-28, 31-33] stratified by sepsis severity, “severe sepsis” cases and controls differed in three models: A vs. G (*P*=0.027, OR=1.55), GA+AA vs. GG (*P*=0.039, OR=1.55), and carrier A vs. carrier G (*P*=0.009, OR=1.30) (Table 2). Figure 2 and Supplementary Figures 1–3 show forest plots of ethnicity subgroup analyses.

### *TNF-α* rs361525 meta-analysis

**Figure 3:**
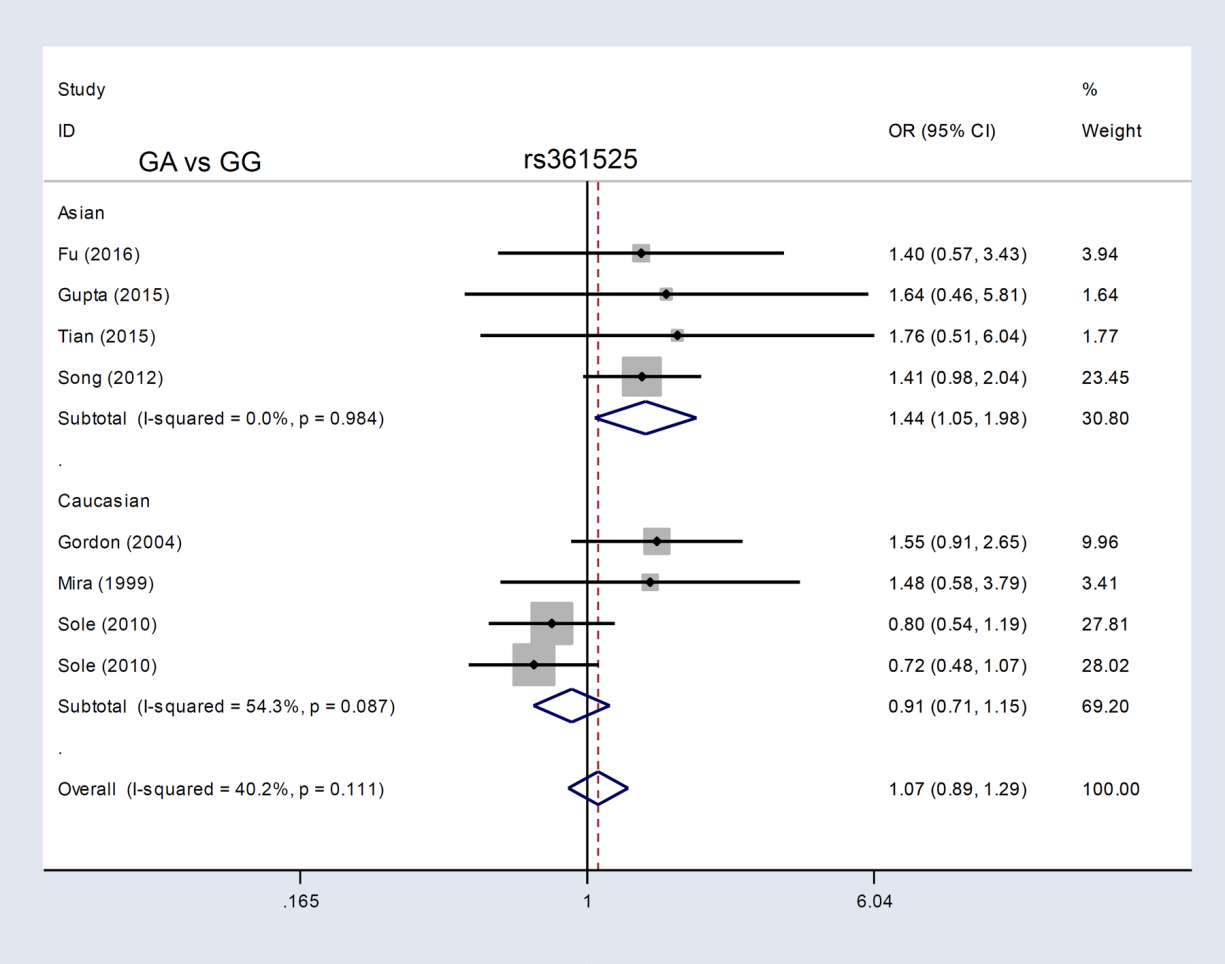
*TNF-α* rs361525 subgroup analysis based on ethnicity using the GA vs. GG genetic model

We enrolled eight case-control studies containing 1,916 cases and 3,372 controls [13, 15, 24, 25, 27, 28, 32, 33] in our *TNF-α* rs361525 meta-analysis. Sepsis risk was increased in the AA vs. GG (*P*=0.001, OR=4.24) and AA vs. GG+GA (*P*=0.001, OR=4.24) genetic models, but not A vs. G, GA vs. GG, GA+AA vs. GG, or carrier A vs. carrier G (all *P*>0.05; Table 3 ). Similarly, for subgroup analyses of sepsis severity, increased risk of severe sepsis or septic shock was only observed in the AA vs. GG and AA vs. GG+GA models (Table 3, all *P*<0.05, OR>1). In contrast, PB subgroup analyses revealed differences in the A vs. G (*P*=0.001, OR=1.52, 95% CI=1.18–1.97), GA vs. GG (*P*=0.006, OR=1.46, 95% CI=1.12–1.91), GA+AA vs. GG (*P*=0.003, OR=1.51, 95% CI=1.15–1.97), and carrier A vs. carrier G (*P*=0.006, OR=1.45, 95% CI=1.11–1.89) models (Table 3). Asian patient subgroup analyses also showed differences in these four models (all *P*<0.05, OR>1). Figure 3 and Supplementary Figures 4–6 show forest plots for ethnicity subgroup analyses. These data suggested that the *TNF-α* rs361525 G/A genotype is associated with enhanced risk of sepsis in the Asian population.

**Table 3:** Genetic relationship between *TNF-α* rs361525 and sepsis risk

Comparison	Subgroup	Sample size		Association test		
		Studies	Case/control	z	*P*_*association*_	OR (95% CI)
**A vs. G**	overall	9	1,916/3,372	1.82	0.069	-
	PB	5	1,214/1,182	3.20	**0.001**	1.52 (1.18–1.97)
	HB	3	382/1,018	0.56	0.574	-
	Caucasian	4	904/2,413	0.49	0.622	-
	Asian	4	975/845	2.68	**0.007**	1.52 (1.12–2.05)
	Severe sepsis	5	817/2,749	0.17	0.863	-
	Septic shock	4	404/2,148	1.33	0.183	-
**AA vs. GG**	overall	6	961/2,552	3.42	**0.001**	4.24 (1.85–9.69)
	PB	3	296/476	1.90	0.058	-
	HB	2	345/904	1.96	0.050	-
	Caucasian	4	904/2,413	2.86	**0.004**	3.81 (1.52–9.52)
	Asian	2	57/139	1.89	0.059	-
	Severe sepsis	3	352/2,037	2.14	**0.032**	3.51 (1.11–11.05)
	Septic shock	4	404/2,148	2.89	**0.004**	4.50 (1.62–12.51)
**GA vs. GG**	overall	9	1,916/3,372	0.53	0.595	-
	PB	5	1,214/1,182	2.77	**0.006**	1.46 (1.12–1.91)
	HB	3	382/1,018	1.66	0.098	-
	Caucasian	4	904/2,413	0.80	0.423	-
	Asian	4	975/845	2.25	**0.024**	1.44 (1.05–1.98)
	Severe sepsis	5	817/2,749	0.95	0.342	-
	Septic shock	4	404/2,148	0.02	0.985	-
**GA+AA vs. GG**	overall	9	1,916/3,372	1.17	0.243	-
	PB	5	1,214/1,182	3.01	**0.003**	1.51 (1.15–1.97)
	HB	3	382/1,018	1.14	0.254	-
	Caucasian	4	904/2,413	0.18	0.858	-
	Asian	4	975/845	2.49	**0.013**	1.49 (1.09–2.05)
	Severe sepsis	5	817/2,749	0.59	0.558	-
	Septic shock	4	404/2,148	0.64	0.519	-
**AA vs. GG+GA**	overall	6	961/2,552	3.41	**0.001**	4.24(1.85–9.72)
	PB	3	296/476	1.83	0.068	-
	HB	2	345/904	1.99	**0.046**	3.35 (1.02–10.99)
	Caucasian	4	904/2,413	2.88	**0.004**	3.87 (1.54–9.69)
	Asian	2	57/139	1.82	0.069	-
	Severe sepsis	3	352/2,037	2.19	**0.029**	3.63 (1.14–11.50)
	Septic shock	4	404/2,148	2.85	**0.004**	4.45 (1.59–12.41)
**carrier A vs. carrier G**	overall	9	1,916/3,372	1.14	0.254	-
	PB	5	1,214/1,182	2.73	**0.006**	1.45 (1.11–1.89)
	HB	3	382/1,018	0.95	0.342	-
	Caucasian	4	904/2,413	0.07	0.948	-
	Asian	4	975/845	2.27	**0.023**	1.44 (1.05–1.97)
	Severe sepsis	5	817/2,749	0.45	0.653	-
	Septic shock	4	404/2,148	0.65	0.517	-

PB: population-based control; HB: hospital-based control; OR: odd ratio; CI: confidence interval; -: ORs (95% CIs) not provided for *P*_association_ >0.05.

### Heterogeneity, publication bias, and sensitivity analysis

**Figure 4:**
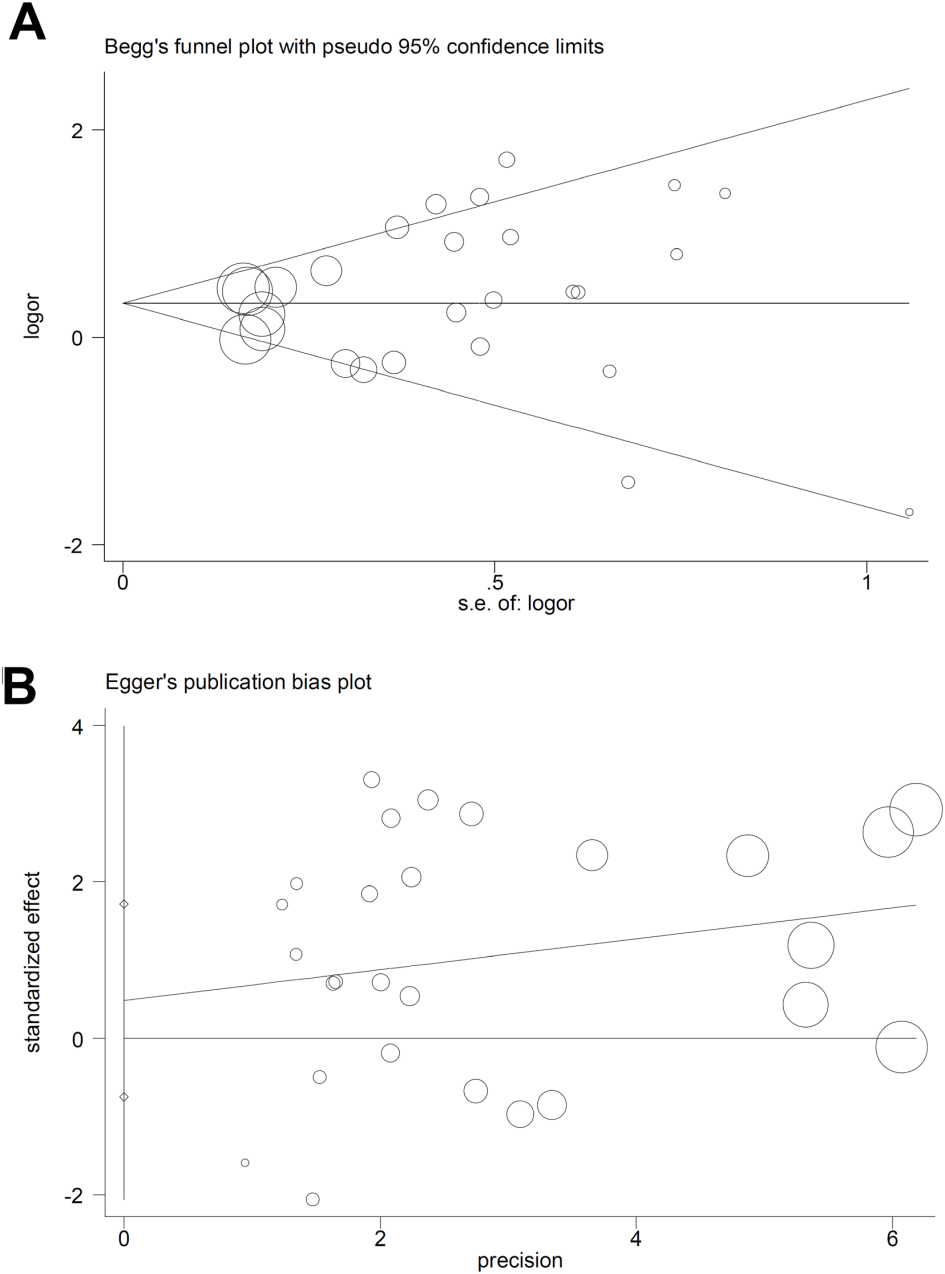
*TNF-α* rs1800629 publication bias analysis using the GA vs. GG genetic model Begg’s funnel plot **(A)** Egger’s publication bias plot **(B)**.

**Figure 5:**
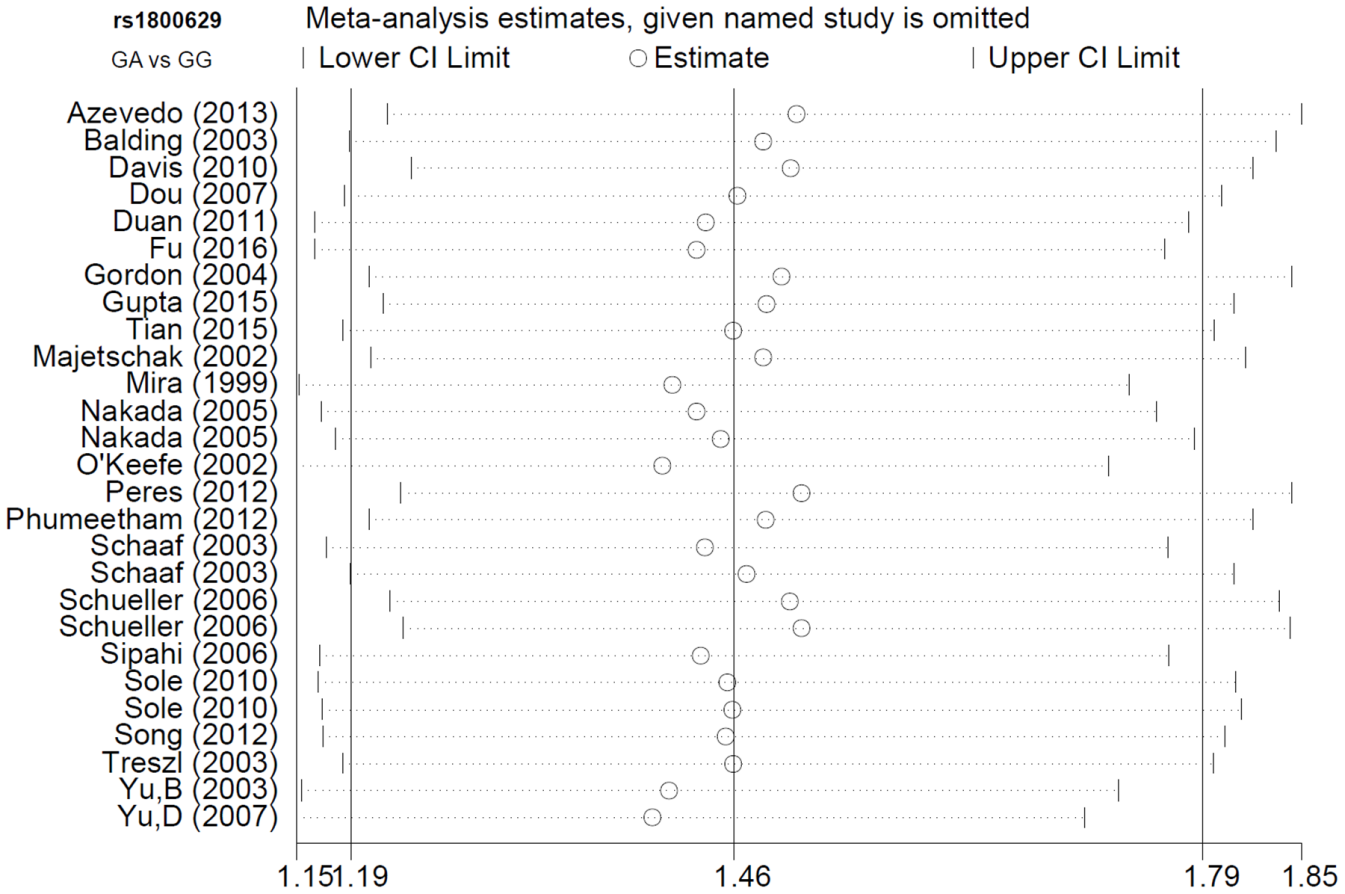
*TNF-α* rs1800629 sensitivity analysis using the GA vs. GG genetic model

For *TNF-α* rs1800629, we applied the random-effect model in the allele, heterozygote, dominant, and carrier Mantel-Haenszel analyses, due to the following data (Table 4): A vs. G (I^2^=55.7%, heterogeneity *P*<0.001); GA vs. GG (I^2^=56.9%, *P*<0.001); GA+AA vs. GG (I^2^=58.2%, *P*<0.001); carrier A vs. carrier G (*P*<0.05). For *TNF-α* rs361525, the fixed-effected model was used for all comparisons (Table 4, all I^2^<50.0%, heterogeneity *P*>0.05). We performed Begg’s test and Egger’s test to evaluate publication bias. We did not observe any large publication bias (*P*>0.05 in both Begg’s test and Egger’s test), except in the rs1800629 Egger’s test in the AA vs. GG (*P*=0.042) and AA vs. GG+GA (*P*=0.041) models (Table 5). Figure 4 shows the Begg’s funnel plot and Egger’s publication bias plot for the GA vs. GG *TNF-α* rs1800629 meta-analysis. Similar pooled ORs in our sensitivity analysis suggested that our data were reliable (Figure 5 for the GA vs. GG model of *TNF-α* rs1800629; other data not shown).

**Table 4:** Heterogeneity evaluation

SNP	Comparison	I^2^	*P*-value	Model
rs1800629	A vs. G	55.7%	**<0.001**	Random
	AA vs. GG	0.0%	0.828	Fixed
	GA vs. GG	56.9%	**<0.001**	Random
	GA+AA vs. GG	58.2%	**<0.001**	Random
	AA vs. GG+GA	0.0%	0.892	Fixed
	carrier A vs. carrier G	37.1%	**0.029**	Random
rs361525	A vs. G	43.6%	0.077	Fixed
	AA vs. GG	0.0%	0.961	Fixed
	GA vs. GG	40.8%	0.095	Fixed
	GA+AA vs. GG	42.3%	0.085	Fixed
	AA vs. GG+GA	0.0%	0.974	Fixed
	carrier A vs. carrier G	27.8%	0.197	Fixed

SNP: single nucleotide polymorphisms.

**Table 5:** Publication bias evaluation

SNP	Comparison	Begg’s test		Egger’s test	
		z	*P*	t	*P*
rs1800629	A vs. G	1.17	0.243	1.68	0.106
	AA vs. GG	1.46	0.144	2.19	**0.042**
	GA vs. GG	0.54	0.588	0.80	0.431
	GA+AA vs. GG	0.92	0.359	1.20	0.243
	AA vs. GG+GA	1.72	0.086	2.20	**0.041**
	carrier A vs. carrier G	1.13	0.260	1.49	0.148
rs361525	A vs. G	0.31	0.754	1.00	0.352
	AA vs. GG	0.00	1.000	1.83	0.141
	GA vs. GG	0.31	0.754	0.44	0.673
	GA+AA vs. GG	-0.10	1.000	0.76	0.469
	AA vs. GG+GA	0.00	1.000	1.59	0.186
	carrier A vs. carrier G	-0.10	1.000	0.72	0.496

SNP: single nucleotide polymorphisms.

## DISCUSSION

This updated literature search and meta-analysis comprehensively reassessed the association between *TNF-α* polymorphisms and sepsis risk in Asian/Caucasian populations. There are several advantages in terms of database searching, screening strategy study inclusion, and sample size. To date, three related meta-analyses have been published [36-38]. Teuffel, *et al.* performed the first of these in 2010, and reported that the GA or AA *TNF-α* rs1800629 genotypes were associated with increased sepsis risk [37]. Twenty-five articles [14, 16, 18, 25-28, 31, 39-55] were included in this meta-analysis, however several articles [39-49, 51-55] did not contain sufficient genotype frequency data in the case and/or control groups, or were not in line with Hardy-Weinberg Equilibrium (HWE). Additionally, no or mild sepsis was set as the control group in one included article [40], which may not have been appropriate for our meta-analysis. Another meta-analysis by Srinivasan, *et al.* only investigated the association between *TNF-α* rs1800629 and neonatal sepsis risk [36]. After rigorous screening, we enrolled 23 articles with 3,404 cases and 5,973 controls [13-35] in our *TNF-α* rs1800629 meta-analysis.

Our study included articles from a variety of databases and we performed statistical analyses using six genetic models. Previous meta-analyses were not performed using different genetic models, and the roles of the GA and AA genotypes were thus not evaluated [37]. Another recent meta-analysis by Zhang, *et al.* assessed 26 articles [13-16, 18, 23-26, 28-32, 34, 41, 44, 47-49, 51, 53, 56-59] and associated both the *TNF-α* rs1800629 and rs361525 polymorphisms with increased sepsis risk [38]. Here, we included only moderate and high quality articles (NOS score >5) from 834 relevant articles from 2007 that contained complete case/control genotype data (GG, GA, AA). Genotype frequency distributions in controls must have been in line with HWE to be included in our analysis. We excluded three articles [41, 44, 57] inconsistent with HWE, and six [47-49, 51, 53, 59] that did not provide sufficient case/control genotype data. We excluded another article [56] that may have been the source of the high heterogeneity in the *TNF-α* rs1800629 Asian patient subgroup analysis. Additionally, our study included eight new articles [17, 19-22, 27, 33, 35] that were not assessed by Zhang, *et al.* [38].

Our stratified analysis of severe sepsis and septic shock included only articles that specified sepsis type. Our subgroup meta-analyses based on controls (HB/PB), showed that the *TNF-α* rs1800629 G/A genotype was linked to increased sepsis risk in both groups. However, *TNF-α* rs361525 and sepsis risk were positively correlated in the PB, but not HB group. Thus, the presence of other diseases may influence the genetic role of *TNF-α* rs361525, but not *TNF-* rs1800629 in sepsis risk.

Our study was subject to certain limitations. First, more studies with larger sample sizes and high qualities are needed for enhanced statistical power. We only observed the potential association between *TNF-α* rs1800629 and severe sepsis risk for the allele, dominant, and carrier models. *TNF-α* rs361525 was also found be slightly linked to the risk of severe sepsis or septic shock for the homozygote and recessive models. Thus, our lack of strong evidence for associations between the two SNPs and sepsis risk merits more case-control studies. Second, we found high heterogeneity between studies in some genetic comparisons. This may be caused at least in part by the complexity of the sepsis etiology and the non-uniformity of diagnostic criteria. Finally, more data are needed to clarify the genetic roles and prognostic significance of distinct cytokine gene combinations in sepsis risk. Different SNP linkages of the *TNF-α* gene should be considered as well.

In conclusion, our findings suggest a positive association between the G/A genotype of the *TNF-α* rs1800629 and rs361525 polymorphisms and sepsis risk in the Asian population, which is partly in line with the findings of Teuffel, *et al.* [37] and Zhang, *et al.* [38]. Abnormal TNF-α is implicated in the pathogenesis of sepsis. For example, *TNF-α* expression was closely related to neonatal sepsis in very low birth weight infants in Spain [60]. It may be that mutation in the *TNF-α* promoter region from common G (guanidine) to rare A (adenosine) at position -308 (rs1800629) or -238 (rs361525) affects normal TNF-α production, secretion, or function in sepsis patients [8].

## MATERIALS AND METHODS

### Records identification

We identified potential records from six databases (PubMed, WOS, EMBASE, CNKI, WANFANG, and Scopus). We performed a PRISMA (preferred reporting items for systematic reviews and meta-analyses [61])-compliant database search and study selection, and the relevant meta-analysis papers were referred [62-64]. Two authors (Yixin Zhang, Xiaoteng Cui) independently removed duplicate studies and assessed record eligibilities according to our exclusion/inclusion criteria. Exclusion criteria included: a) meta-analysis; b) review, editorials, and perspectives; c) congress abstract or poster; d) other gene or other disease; e) not a *TNF-α* SNP or no clinical data; f) lack of control data; g) did not contain genotype data or the genotype distributions were not in line with HWE.

According to Newcastle-Ottawa Scale (NOS) requirements (http://www.ohri.ca/programs/clinical_epidemiology/oxford.asp), we evaluated methodological quality independently. Included studies had a NOS score >5, and provide the following data: first author name, publication year, patient ethnicity, SNP, sample size, and genotype frequency within case-control studies and genotyping assays.

### Quantitative synthesis and heterogeneity

We used the Mantel-Haenszel test to determine *P*-values, pooled ORs, and 95% CIs for the following six genetic models: A vs. G (allele); AA vs. GG (homozygote); GA vs. GG (heterozygote); GA+AA vs. GG (dominant); AA vs. GG+GA (recessive); and carrier A vs. carrier G (carrier). *P*<0.05 represented a statistically significant difference between case and control studies. We also assessed the heterogeneity between studies using the Q statistic and I^2^ test. High heterogeneity was likely when Q statistic *P*<0.05 or I^2^>50%. In this situation, we employed the random-effect model, not the fixed-effect model. We also performed a series of subgroup meta-analyses according to three factors: source of control [PB (population-based) or HB (hospital-based)], ethnicity (Caucasian or Asian), and sepsis severity (sepsis, severe sepsis, or septic shock).

### Publication bias and sensitivity analysis

We evaluated publication bias using both Begg’s test and Egger’s test. *P*>0.05 in both tests would exclude a large publication bias. We also performed a sensitivity analysis to evaluate the robustness of our data. All analyses were performed using Stata/SE 12.0 software (StataCorp, USA).

## SUPPLEMENTARY MATERIALS FIGURES AND TABLES






