# Supplementary material for: The effects of tumor necrosis factor-α (TNF-α) rs1800629 and rs361525 polymorphisms on sepsis risk
Source: Oncotarget. 2017 Nov 30;8(67):111456–69. doi: 10.18632/oncotarget.22824 (PMC5762335; doi:10.18632/oncotarget.22824)
Supplement: Supplementary file 1 [file oncotarget-08-111456-s001.pdf]

The effects of tumor necrosis factor-α (TNF-α) rs1800629 and rs361525 polymorphisms on sepsis risk

SUPPLEMENTARY MATERIALS

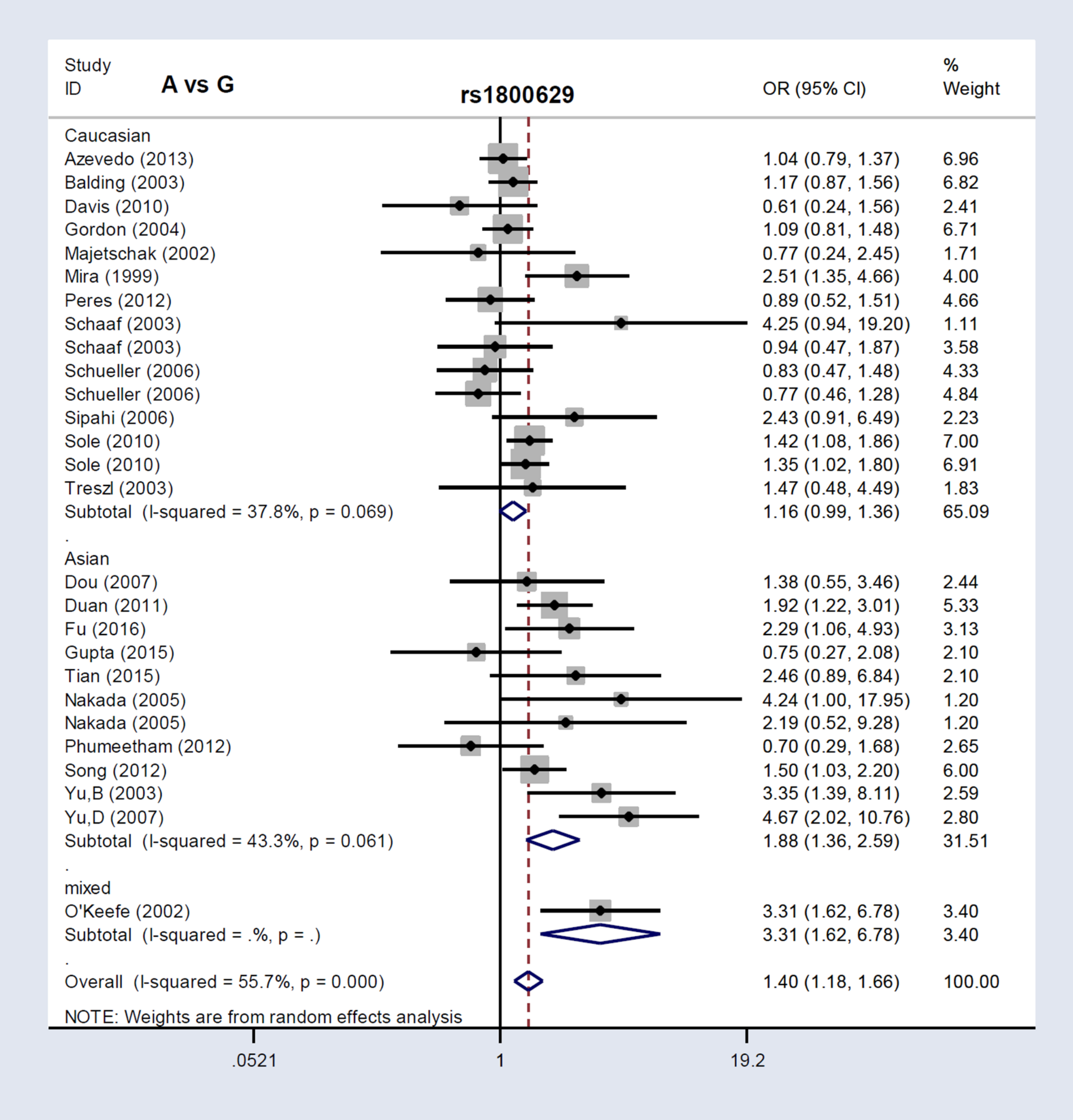

Supplementary Figure 1: ***TNF-α*** **rs1800629 subgroup analysis based on ethnicity using the A vs. G genetic model.**

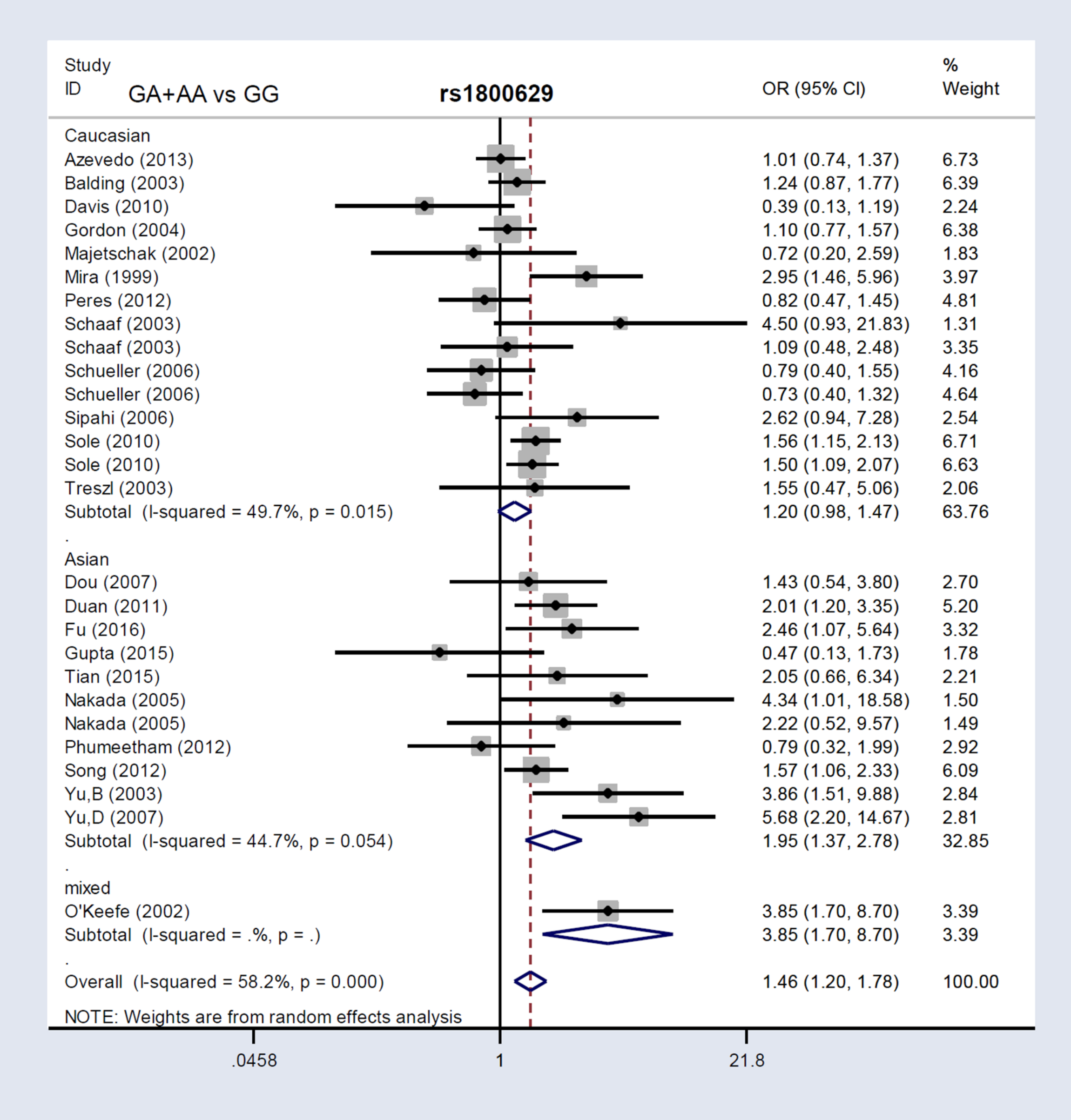

Supplementary Figure 2: ***TNF-α*** **rs1800629 subgroup analysis based on ethnicity using the GA+AA vs. GG genetic model.**

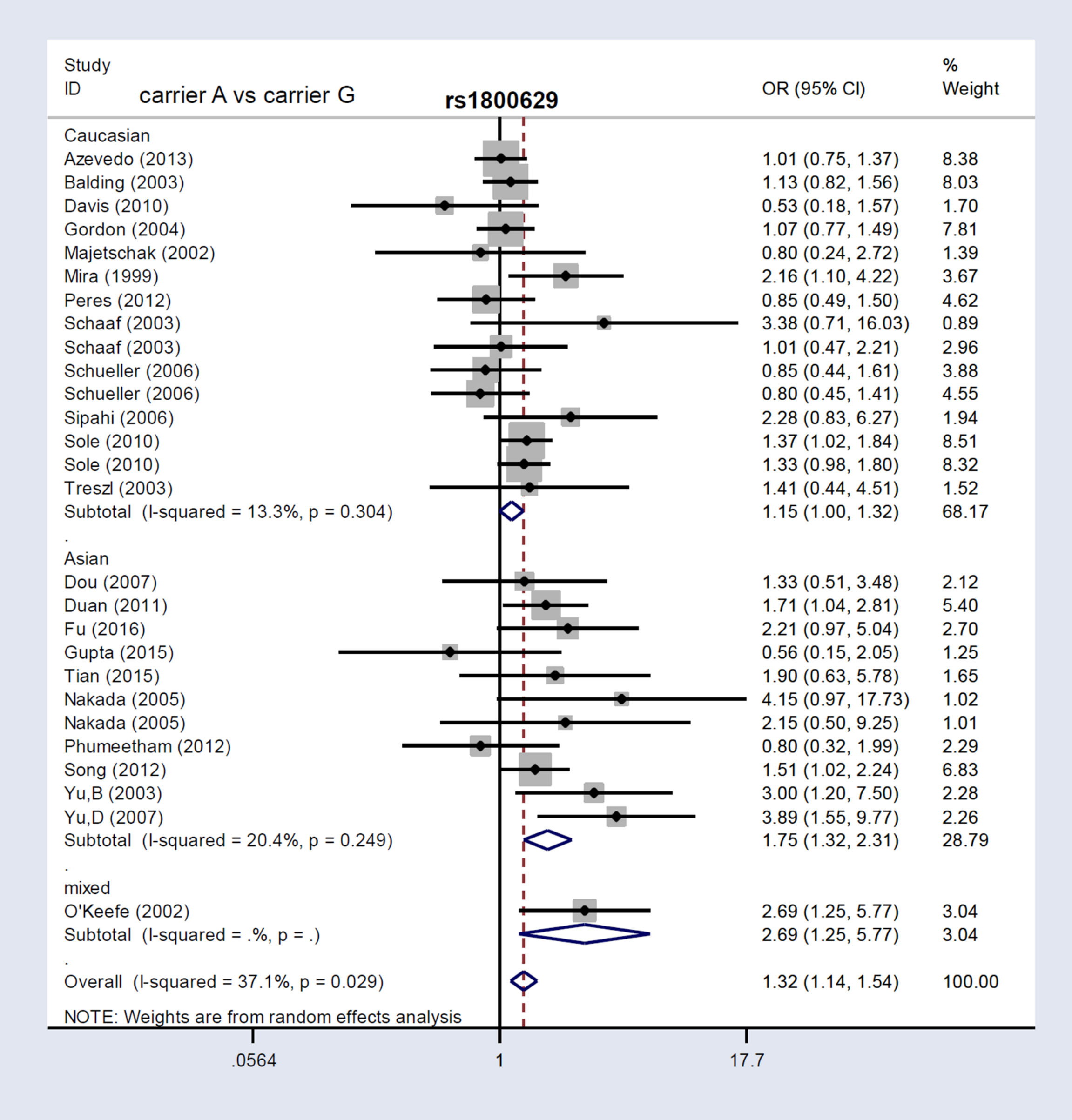

Supplementary Figure 3: ***TNF-α*** **rs1800629 subgroup analysis based on ethnicity using the carrier A vs. carrier G genetic model.**

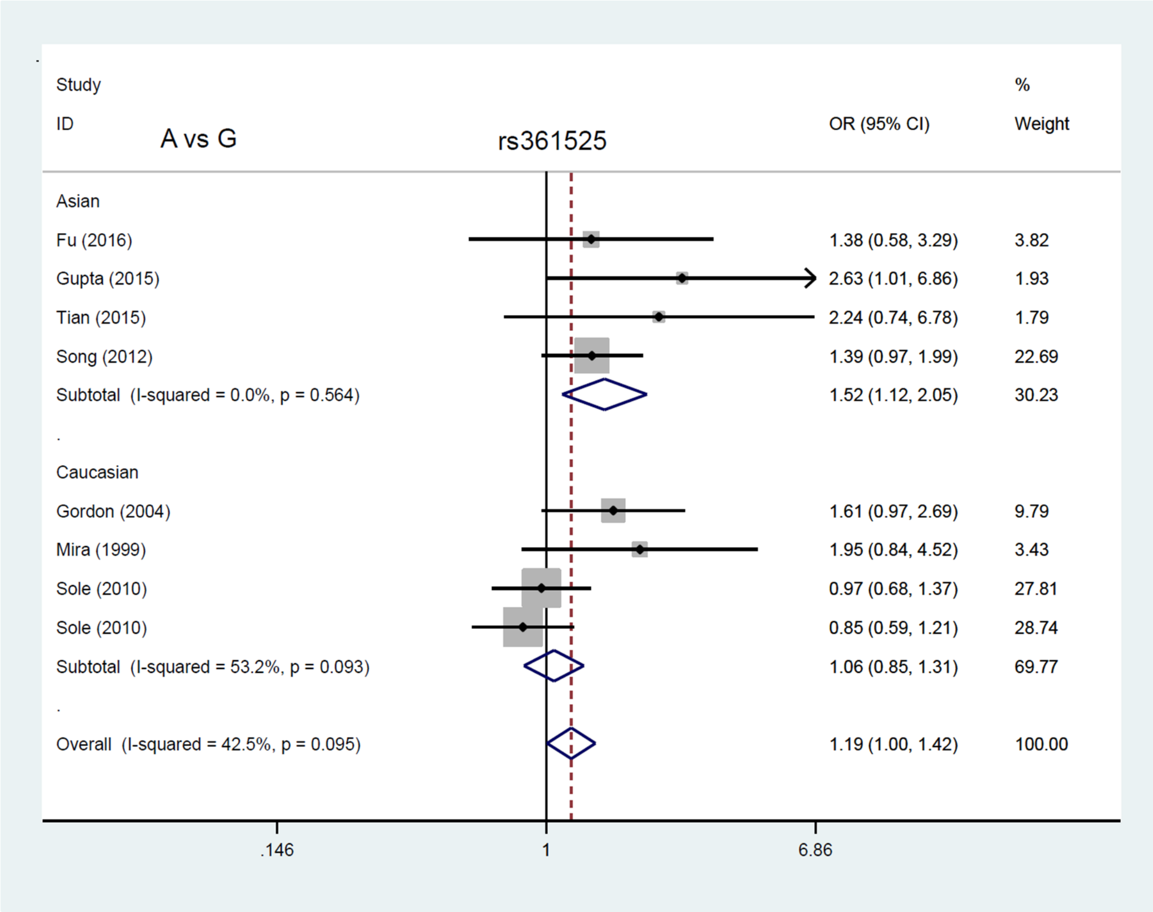

Supplementary Figure 4: *TNF-α* rs361525 subgroup analysis based on ethnicity using the A vs. G genetic model.

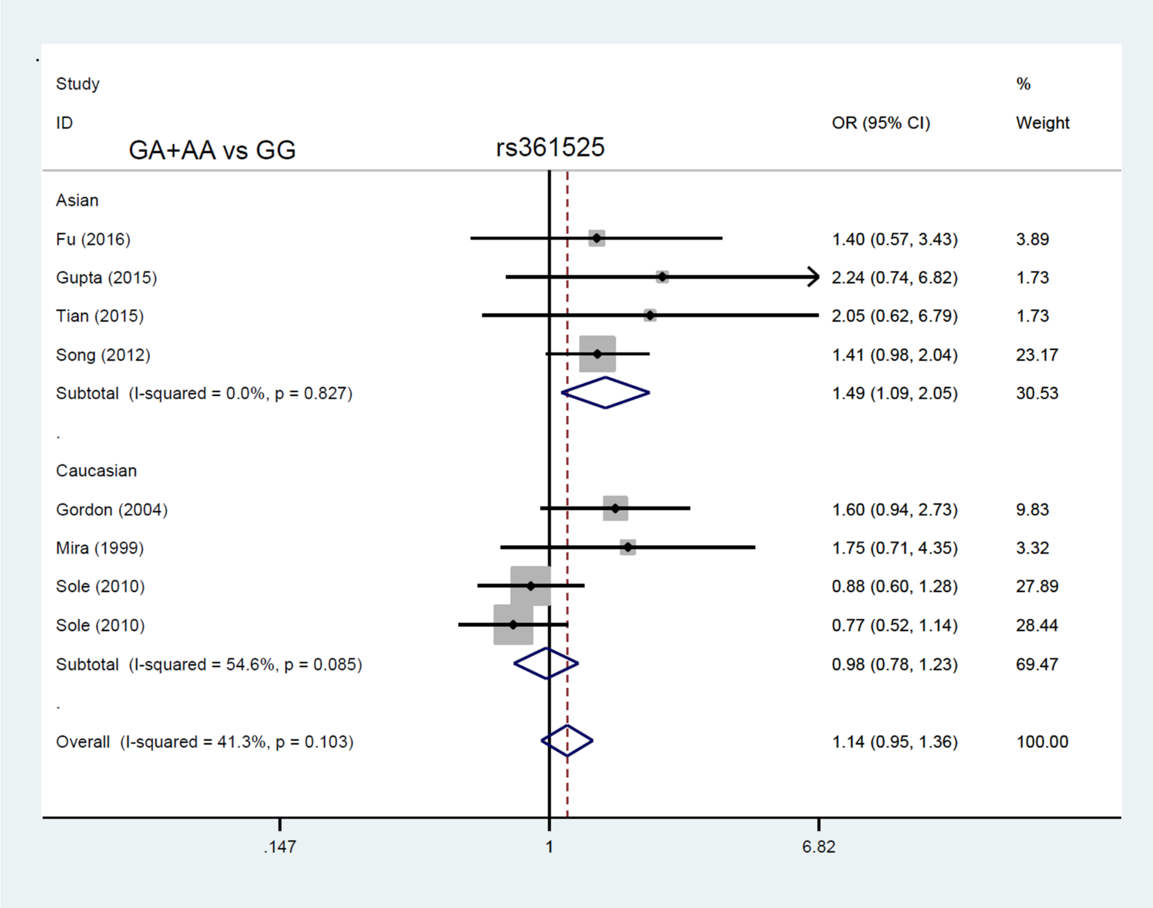

Supplementary Figure 5: *TNF-α* rs361525 subgroup analysis based on ethnicity using the GA+AA vs. GG genetic model.

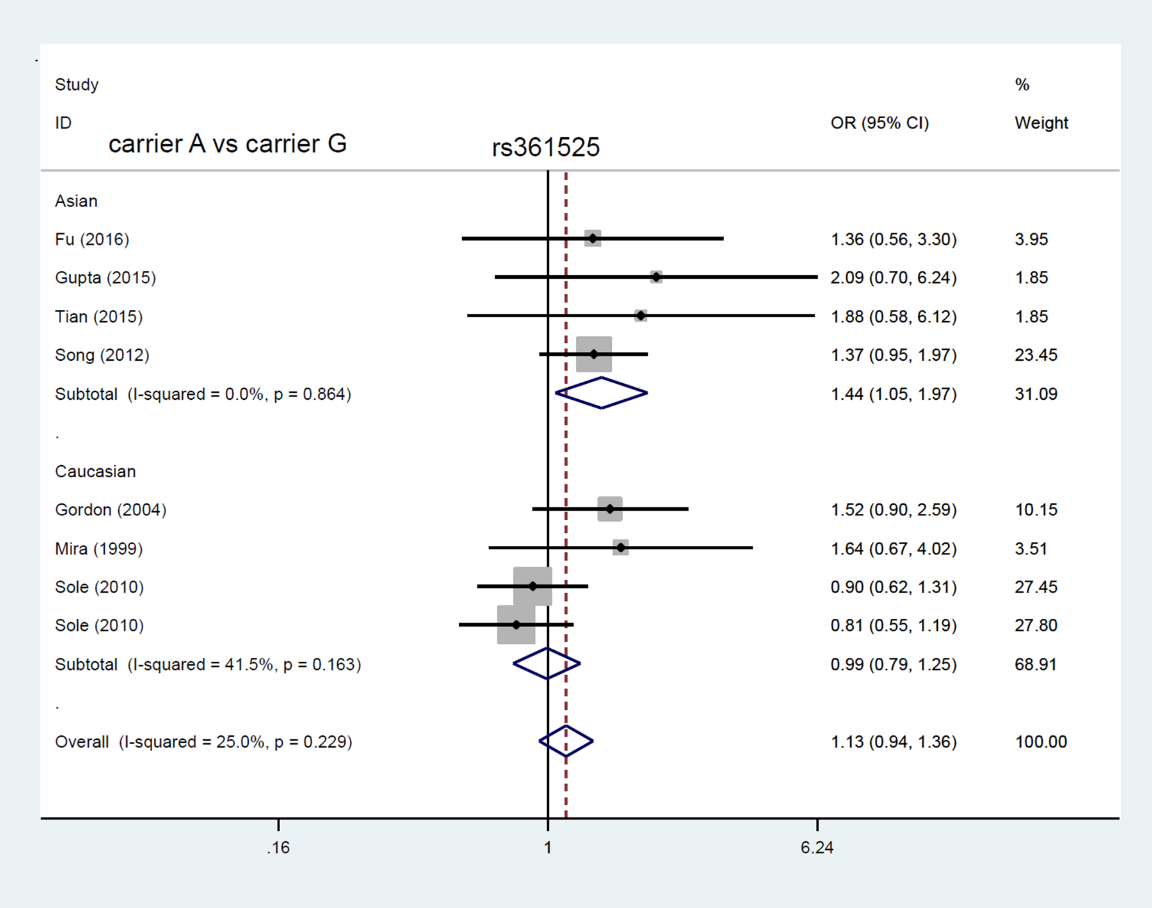

Supplementary Figure 6: ***TNF-α*** **rs361525 subgroup analysis based on ethnicity using the carrier A vs. carrier G genetic model.**

Supplementary Table 1: Database search strategy

See Supplementary File 1

Supplementary Table 2: Case/control group genotype frequencies

See Supplementary File 2
