# Supplementary material for: The effects of tumor necrosis factor-α (TNF-α) rs1800629 and rs361525 polymorphisms on sepsis risk
Source: Oncotarget. 2017 Nov 30;8(67):111456–69. doi: 10.18632/oncotarget.22824 (PMC5762335; doi:10.18632/oncotarget.22824)
Supplement: Supplementary file 2 [file oncotarget-08-111456-s002.docx]

Supplementary Table 1: Database search strategy.

| 1. **PubMed** | | |
| --- | --- | --- |
| **Step** | **Research terms** | **Article number** |
| #1 | "Sepsis"[Mesh] | 105,444 |
| #2 | (((((((((((((Severe Sepsis) OR Sepsis, Severe) OR Pyemia) OR Pyemias) OR Pyohemia) OR Pyohemias) OR Pyaemia) OR Pyaemias) OR Septicemia) OR Septicemias) OR Poisoning, Blood) OR Blood Poisoning) OR Blood Poisonings) OR Poisonings, Blood | 200,408 |
| #3 | (#1 OR #2) | 200,408 |
| #4 | "Tumor Necrosis Factor-alpha"[Mesh] | 108,167 |
| #5 | ((((((((Tumor Necrosis Factor alpha) OR Cachectin-Tumor Necrosis Factor) OR Cachectin Tumor Necrosis Factor) OR TNFalpha) OR TNF-alpha) OR Tumor Necrosis Factor) OR Tumor Necrosis Factor Ligand Superfamily Member 2) OR Cachectin) OR TNF Superfamily, Member 2 | 226,236 |
| #6 | (#4 OR #5) | 226,236 |
| #7 | (#3 AND #6) | 8,997 |
| #8 | "Polymorphism, Single Nucleotide"[Mesh] | 87,156 |
| #9 | (((((Nucleotide Polymorphism, Single) OR Nucleotide Polymorphisms, Single) OR Polymorphisms, Single Nucleotide) OR Single Nucleotide Polymorphisms) OR SNPs) OR Single Nucleotide Polymorphism | 113,554 |
| #10 | (#8 OR #9) | 113,554 |
| #11 | (#7 AND #10) | 96 |
| 1. **WOS** | | |
| #1 | TOPIC: (Sepsis) OR TOPIC: (Severe Sepsis) OR TOPIC: (Sepsis, Severe) OR TOPIC: (Pyemia) OR TOPIC: (Pyemias) OR TOPIC: (Pyohemia) OR TOPIC: (Pyohemias) OR TOPIC: (Pyaemia) OR TOPIC: (Pyaemias) OR TOPIC: (Septicemia) OR TOPIC: (Septicemias) OR TOPIC: (Poisoning, Blood) OR TOPIC: (Blood Poisoning) OR TOPIC: (Blood Poisonings) OR TOPIC: (Poisonings, Blood)  Timespan=All years  Search language=Auto | 245,849 |
| #2 | TOPIC: (Tumor Necrosis Factor-alpha) OR TOPIC: (Tumor Necrosis Factor alpha) OR TOPIC: (Cachectin-Tumor Necrosis Factor) OR TOPIC: (Cachectin Tumor Necrosis Factor) OR TOPIC: (TNFalpha) OR TOPIC: (TNF-alpha) OR TOPIC: (Tumor Necrosis Factor) OR TOPIC: (Tumor Necrosis Factor Ligand Superfamily Member 2) OR TOPIC: (TNF Superfamily, Member 2)  Timespan=All years  Search language=Auto | 335,552 |
| #3 | TOPIC: (Polymorphism, Single Nucleotide) OR TOPIC: (Nucleotide Polymorphism, Single) OR TOPIC: (Nucleotide Polymorphisms, Single) OR TOPIC: (Polymorphisms, Single Nucleotide) OR TOPIC: (Single Nucleotide Polymorphisms) OR TOPIC: (SNPs) OR TOPIC: (Single Nucleotide Polymorphism)  Timespan=All years  Search language=Auto | 162,095 |
| #4 | (#3 AND #2 AND #1) | 182 |
| 1. **EMBASE** | | |
| #1 | 'sepsis'/exp | 212,200 |
| #2 | 'abdominal sepsis' | 1,842 |
| #3 | 'focal sepsis' | 37 |
| #4 | 'intraabdominal sepsis' | 333 |
| #5 | 'sepsis syndrome' | 1,068 |
| #6 | 'septic disease' | 138 |
| #7 | (#1 OR #2 OR #3 OR #4 OR #5 OR #6) | 212,955 |
| #8 | 'tumor necrosis factor' | 288,993 |
| #9 | 'cachectin' | 475 |
| #10 | 'cachetin' | 18 |
| #11 | 'human recombinant tumour necrosis factor alpha' | 11 |
| #12 | 'mhr 24' | 5 |
| #13 | 'recombinant tumour necrosis factor alpha' | 33 |
| #14 | 'tissue necrosis factor' | 197 |
| #15 | 'tnf alfa' | 673 |
| #16 | 'tnf alpha' | 17,470 |
| #17 | 'tumor necrosis factor alfa' | 319 |
| #18 | 'tumor necrosis factor alpha' | 35,660 |
| #19 | 'tumor necrosis factor-alpha; tumor necrosis factors' | 0 |
| #20 | 'tumor necrosis serum; tumour necrosis factor' | 0 |
| #21 | 'tumour necrosis factor alfa' | 29 |
| #22 | 'tumour necrosis factor alpha' | 4,619 |
| #23 | 'tumour necrosis factor-alpha' | 4,619 |
| #24 | 'tumour necrosis factors' | 109 |
| #25 | 'tumour necrosis serum' | 8 |
| #26 | (#8 OR #9 OR #10 OR #11 OR #12 OR #13 OR #14 OR #15 OR #16 OR #17 OR #18 OR #19 OR #20 OR #21 OR #22 OR #23 OR #24 OR #25) | 293,390 |
| #27 | 'single nucleotide polymorphism' | 135,249 |
| #28 | 'polymorphism, single nucleotide' | 362 |
| #29 | (#27 OR #28) | 135,347 |
| #30 | (#7 AND #26 AND #29) | 180 |
| 1. **CNKI** | | |
| #1 | SU='脓毒症' and SU='肿瘤坏死因子' and SU='基因多态性' | 20 |
| 1. **WANFANG** | | |
| #1 | 脓毒症 * 肿瘤坏死因子 * 基因多态性 * Date:-2017 | 26 |
| 1. **Scopus** | | |
| #1 | TITLE-ABS-KEY ( "Sepsis" OR "Severe Sepsis" OR "Septic shock" OR "Pyemia" OR "Pyohemia" OR "Pyohemias" OR "Pyaemia" OR "Pyaemias" OR "Septicemia" OR "Blood Poisoning" ) | 195,824 |
| #2 | TITLE-ABS-KEY ( "Tumor Necrosis Factor-alpha" OR "Cachectin-Tumor Necrosis Factor" OR "TNFalpha" OR "TNF-alpha" OR "Tumor Necrosis Factor Ligand Superfamily Member 2" OR "TNF-α" OR "TNF Superfamily, Member 2" ) | 235,285 |
| #3 | TITLE-ABS-KEY ( "Polymorphism, Single Nucleotide" OR "Polymorphisms" OR "Polymorphism" OR "Single Nucleotide Polymorphisms" OR "SNPs" OR "Single Nucleotide Polymorphism" ) | 423,886 |
| #4 | (#1 AND #2 AND #3) | 330 |
