# Supplementary material for: The effects of tumor necrosis factor-α (TNF-α) rs1800629 and rs361525 polymorphisms on sepsis risk
Source: Oncotarget. 2017 Nov 30;8(67):111456–69. doi: 10.18632/oncotarget.22824 (PMC5762335; doi:10.18632/oncotarget.22824)
Supplement: Supplementary file 3 [file oncotarget-08-111456-s003.docx]

Supplementary Table 2: Case/control group genotype frequencies.

| **First author, year** | **SNP** | **GG/GA/AA** | **Case note** | **GG/GA/AA** | **Control note** | **Source of control** | **HWE** |
| --- | --- | --- | --- | --- | --- | --- | --- |
| Azevedo, 2012 | rs1800629 | 348/80/11 | ARDS with sepsis | 448/105/11 | healthy children | PB | 0.105 |
| Balding, 2003 | rs1800629 | 100/75/8 | meningococcal sepsis | 233/140/16 | Caucasian Irish blood donors | HB | 0.375 |
| Davis, 2010 | rs1800629 | 23/3/2 | puerperal group A streptococcal sepsis | 34/18/1 | racially matched and uncomplicated deliveries | PB | 0.426 |
| Dou, 2007 | rs1800629 | 35/10/0 | sepsis with different causes | 50/10/0 | normal control | PB | 0.481 |
| Duan, 2011 | rs1800629 | 86/39/6 | major trauma with sepsis | 138/33/3 | major trauma without sepsis | HB | 0.535 |
| Fu, 2016 | rs1800629 | 94/19/2 | sepsis patients with a history of chronic heart, renal, liver or pulmonary failure, including trauma subjects. | 99/8/1 | healthy individuals | PB | 0.094 |
|  | rs361525 | 102/13/0 | sepsis patients with a history of chronic heart, renal, liver or pulmonary failure, including trauma subjects | 99/9/0 | healthy individuals | PB | 0.651 |
| Gordon, 2004 | rs1800629 | 135/69/8 | severe sepsis/septic shock | 233/110/11 | normal health controls | PB | 0.647 |
|  | rs361525 | 176/28/1 | severe sepsis/septic shock | 321/33/0 | normal health controls | PB | 0.358 |
| Gupta, 2015 | rs1800629 | 22/1/2 | trauma hemorrhagic shock with sepsis | 69/17/3 | trauma hemorrhagic shock without sepsis | HB | 0.154 |
|  | rs361525 | 19/4/2 | trauma hemorrhagic shock with sepsis | 78/10/1 | trauma hemorrhagic shock without sepsis | HB | 0.315 |
| Majetschak, 2002 | rs1800629 | 10/4/0 | blunt trauma with severe sepsis | 36/20/0 | trauma patients without severe sepsis | HB | 0.104 |
| Mira, 1999 | rs1800629 | 46/32/3 | septic shock | 62/15/1 | healthy unrelated blood donors | PB | 0.931 |
|  | rs361525 | 46/11/2 | septic shock | 62/10/0 | healthy unrelated blood donors | PB | 0.527 |
| Nakada, 2005 | rs1800629 | 81/5/0 | critically ill patients with sepsis | 211/3/0 | healthy Japanese volunteers | PB | 0.918 |
|  | rs1800629 | 81/5/0 | critically ill patients with sepsis | 108/3/0 | critically ill patients without sepsis | HB | 0.885 |
| O'Keefe, 2002 | rs1800629 | 21/15/1 | trauma patients with severe sepsis | 96/19/0 | trauma patients without severe sepsis | HB | 0.334 |
|  | rs361525 | 36/1/0 | trauma patients with severe sepsis | 103/11/0 | trauma patients without severe sepsis | HB | 0.588 |
| Peres, 2012 | rs1800629 | 143/21/2 | sepsis | 179/34/1 | volunteer subjects | PB | 0.648 |
| Phumeetham, 2012 | rs1800629 | 58/8/0 | pediatric patients with sepsis/septic shock | 86/13/2 | healthy controls | PB | 0.097 |
| Schaaf, 2003 | rs1800629 | 17/11/0 | pneumococcal sepsis | 16/2/0 | pneumococcal disease without sepsis | HB | 0.803 |
|  | rs1800629 | 7/2/1 | pneumococcal severe sepsis | 16/2/0 | pneumococcal disease without sepsis | HB | 0.803 |
|  | rs1800629 | 8/3/1 | pneumococcal septic Shock | 16/2/0 | pneumococcal disease without sepsis | HB | 0.803 |
|  | rs1800629 | 17/11/0 | pneumococcal sepsis | 33/13/4 | age-matched control subjects | HB | 0.126 |
|  | rs1800629 | 7/2/1 | pneumococcal severe sepsis | 33/13/4 | age-matched control subjects | HB | 0.126 |
|  | rs1800629 | 8/3/1 | pneumococcal septic Shock | 33/13/4 | age-matched control subjects | HB | 0.126 |
| Schueller, 2006 | rs1800629 | 48/16/3 | 67 premature infants <32 weeks of gestational age with proven early-onset sepsis | 68/29/5 | 102 healthy newborn infants | PB | 0.415 |
|  | rs1800629 | 48/16/3 | 67 premature infants <32 weeks of gestational age with proven early-onset sepsis | 151/69/13 | 233 healthy adult volunteers | PB | 0.181 |
| Sipahi, 2006 | rs1800629 | 42/11/0 | severe sepsis | 70/7/0 | healthy volunteers | PB | 0.676 |
| Sole, 2010 | rs1800629 | 124/35/1 | CAP with sepsis shock | 870/260/22 | unrelated healthy volunteers+patient without a previous history of relevant infectious diseases | PB+HB | 0.617 |
|  | rs1800629 | 122/35/3 | CAP with severe sepsis | 870/260/22 | unrelated healthy volunteers+patient without a previous history of relevant infectious diseases | PB+HB | 0.617 |
|  | rs361525 | 138/20/2 | CAP with sepsis shock | 1016/153/3 | unrelated healthy volunteers+patient without a previous history of relevant infectious diseases | PB+HB | 0.127 |
|  | rs361525 | 144/14/2 | CAP with severe sepsis | 1016/153/3 | unrelated healthy volunteers+patient without a previous history of relevant infectious diseases | PB+HB | 0.127 |
|  | rs1800629 | 124/35/1 | CAP with sepsis shock | 610/188/18 | CAP without sepsis | HB | 0.437 |
|  | rs1800629 | 122/35/3 | CAP with severe sepsis | 610/188/18 | CAP without sepsis | HB | 0.437 |
|  | rs361525 | 138/20/2 | CAP with sepsis shock | 694/117/4 | CAP without sepsis | HB | 0.695 |
|  | rs361525 | 144/14/2 | CAP with severe sepsis | 694/117/4 | CAP without sepsis | HB | 0.695 |
| Song, 2012 | rs1800629 | 352/23/0 | sepsis | 560/38/2 | healthy controls | PB | 0.126 |
|  | rs1800629 | 369/56/2 | severe sepsis | 560/38/2 | healthy controls | PB | 0.126 |
|  | rs361525 | 333/42/0 | sepsis | 550/48/0 | healthy controls | PB | 0.307 |
|  | rs361525 | 382/46/0 | severe sepsis | 550/48/0 | healthy controls | PB | 0.307 |
| Tian, 2015 | rs1800629 | 24/6/2 | severe sepsis | 43/7/0 | healthy controls | PB | 0.595 |
|  | rs361525 | 25/6/1 | severe sepsis | 44/6/0 | healthy controls | PB | 0.652 |
| Treszl, 2003 | rs1800629 | 25/8/0 | VLBW neonates with sepsis | 29/6/0 | healthy VLBW neonates | PB | 0.579 |
| Yu,B, 2003 | rs1800629 | 28/12/0 | sepsis | 90/10/0 | healthy controls | PB | 0.599 |
| Yu,D, 2007 | rs1800629 | 32/20/4 | sepsis | 53/6/1 | healthy controls | PB | 0.128 |

SNP: single nucleotide polymorphisms; ARDS: Acute respiratory distress syndrome; CAP: community-acquired pneumonia; VLBW: very-low-birth-weight; HWE: Hardy-Weinberg equilibrium; PB: population-based control; HB: hospital-based control.
